# Telehealth mitigates COPD disease progression compared to standard of care: a randomized controlled crossover trial

**DOI:** 10.1111/joim.13230

**Published:** 2021-01-11

**Authors:** F. Rassouli, A. Germann, F. Baty, M. Kohler, D. Stolz, R. Thurnheer, T. Brack, C. Kähler, S. Widmer, U. Tschirren, N. A. Sievi, M. Tamm, M. H. Brutsche

**Affiliations:** ^1^ From the Lung Center Cantonal Hospital St. Gallen St. Gallen Switzerland; ^2^ Clinic for Pulmonology University Hospital Zurich Zurich Switzerland; ^3^ Clinic for Pulmonology University Hospital Basel Basel Switzerland; ^4^ Clinic for Internal Medicine Cantonal Hospital Münsterlingen Münsterlingen Switzerland; ^5^ Clinic for Internal Medicine Cantonal Hospital Glarus Glarus Switzerland; ^6^ Clinic for Pulmonology Waldburg‐Zeil‐Kliniken Wangen Germany

**Keywords:** COPD, COPD assessment test, COPD exacerbation, telehealth

## Abstract

**Background:**

We showed excellent adherence and satisfaction with our telehealth care (TC) approach for COPD. Here, the results of a consecutive randomized controlled trial are presented.

**Methods:**

Patients were randomly assigned to TC or standard care (SC). During TC, patients answered six daily questions online, and focused on the early recognition of exacerbations, in addition to SC.

**Results:**

The mean increase in COPD assessment test (CAT) was 1.8 vs. 3.6 points/year in the TC and SC groups, respectively (*P* = 0.0015). Satisfaction with care (VAS) at baseline was 8.2; at the end of SC, 8.5 (*P* = 0.062); and after TC, 8.8 (*P* < 0.001). We detected significantly more moderate exacerbations during TC.

**Conclusion:**

Whilst receiving TC, the slope of the CAT increase – an indicator of the naturally progressive course of COPD – was reduced by 50%. Satisfaction with care increased with TC. The higher number of detected moderate exacerbations probably indicates a higher diagnostic sensitivity than without TC.

## Introduction

Telehealth care (TC) could be a relevant component of integrated care for chronic obstructive pulmonary disease (COPD). In brief, TC was described by McLean [1] as electronic transfer of information from the patient over a distance to healthcare professionals, who give personalized feedback and advice to the patient. By the current state, there are still conflicting results considering the improvement of health‐related quality of life (HRQOL) or reduction in acute exacerbations of COPD (AECOPD) by TC [1, 2, 3, 4]. In addition, there exists a wide variability of different interventions in TC, such as monitoring of physiological parameters and vital signs or self‐reported symptoms [5]. It remains unclear which modalities should best be used to improve the patient’s health status or to detect exacerbations [6, 7].

AECOPD is characterized by symptoms, which often occur already days before the event [8]. For early detection of such exacerbation symptoms, disease‐specific questions can be used. In a pilot study, 6 questions were successfully used for the early detection of AECOPD. Further, the feasibility of the same TC procedure as used in this study was shown. Patients appreciated TC and satisfaction with care improved [9]. An extended analysis of this pilot study investigated the change in the COPD assessment test (CAT) score. The slope of the CAT increase was positively correlated with the risk of future exacerbations [10].

The primary aim of the current study was to investigate the impact of a TC procedure on the course of COPD and HRQOL as assessed by the slope of individual CAT changes over the study periods.

## Materials and methods

### Trial design

This was a multicentre randomized controlled crossover trial, involving 6 centres in Switzerland and Germany. Inclusion period was from April 2016 to September 2018. Last patient last visit was September 2019.

### Patients

The continuous screening of all COPD patients took place in daily clinical practice. Each patient participated in the study for 12 months with a crossover after 6 months (Figure S1). Inclusion criteria were a diagnosis of COPD and age ≥ 40 years. The only exclusion criteria were inability to provide informed consent or to follow trial procedures. Trial language was German. All patients provided written informed consent. The study was approved by the Swiss Institutional Review Board (Swissethics, EKSG 15/184, BASEC Nr. 2015‐00065). Trial registration number at ClinicalTrials.gov is NCT04485832.

### General procedure

After screening and inclusion, a first consultation was arranged to collect baseline characteristics, perform randomization and give instructions to the participants. A user account on a web‐based online healthcare platform was installed (‘Evita’ by Swisscom) on the patient’s own technical device. For the study, an adapted plug‐in was created. In the following 6 months, patients starting with the intervention phase daily answered six questions, which were focused on the detection of AECOPD, and completed the CAT weekly. Patients starting with the control phase only filled the CAT questionnaire weekly. After 6 months, patients presented for the second visit, where the crossover took place. The last visit took place after 12 months. During the whole year, all patients had consultations for their COPD treatment as usual. Same access for both groups to equal clinical care according to local practice was granted.

### TC intervention

The daily asked ‘yes’ or ‘no’ questions focused on the recognition of AECOPD (Figure S2). Whilst being in the intervention phase, patients had to answer them every morning. One reminder short message was automatically sent in the late morning in the case of missing entry. From Monday to Friday, the study team analysed all answers in the late morning. An overview (‘cockpit’) showed all patients and for each day of the past week one small box. The different colours of the boxes indicated whether (i) the patient had not answered the questions (grey), (ii) had answered zero or one question with ‘yes’ (green), (iii) had answered two or more questions with ‘yes’, but had a green box on the previous day (yellow), or (iv) had answered two or more questions with ‘yes’ and already had a yellow or red box the previous day (red) (Figure S2). The study team reacted according to a prespecified action plan. A yellow box was a first warning sign, but no action was carried out. If the following day the box turned to red (=two ‘yes’ answers on two consecutive days), this indicated a possible AECOPD. The patient was then contacted by phone by the study team for further evaluation. From that moment on, the physician performing the phone call assumed responsibility for all further actions taken. This could mean to instruct the patient to administer self‐medication, to consult the general practitioner (GP) or to visit the ambulatory or the emergency department (ED) for clinical judgement.

### HRQOL measurement

The CAT was filled weekly by the patients in both phases. For the study, the German version of the questionnaire was used.

### End‐points

The primary end‐point was the group‐specific slope difference in weekly CAT over time. Satisfaction with care was assessed by a visual analogue scale from 0 to 10. Additional end‐points were the number of exacerbations and hospitalizations (as well as the days in hospital) due to AECOPD and the treatment costs per patient and year. Data were collected through hospital records, contacting the patient’s general practitioner and the rehabilitation clinics and asking the patients themselves. Treatment costs included the exact expenses for hospital and rehabilitation stays as well as estimated costs for planned and unplanned outpatient visits, medication and telephone contacts.

### Statistical considerations

The evolution of the CAT over time was analysed using linear mixed‐effects models. The fixed‐effects terms included time, group (intervention vs. control) and the sequential period in the crossover design. The patient ID was defined as a random‐effects term. The secondary and exploratory end‐points were analysed using generalized linear mixed models (including logistic and Poisson regression) and Student's paired t‐tests. All parameter estimates were reported together with their 95% confidence intervals. All analyses were done using the R statistical software with the extension package lme4.

### Sample size calculation

The trial was designed to detect a clinically relevant within‐patient difference in 2 CAT score points in 1 year. In order to detect a statistically significant difference in the primary end‐point in a within‐patient crossover design with a power of 80% and a significance level of 5% (two‐sided), and considering an expected dropout rate of 5% and a learning effect explaining 25% of the effect size in the patients starting with the intervention phase, 175 patients should be included in the study.

## Results

In total, 150 of 168 patients (89%) completed the trial. The baseline characteristics of all 168 included patients are summarized in Table 1.

**Table 1:** Baseline characteristics

Number of patients included	168
Median age, years (IQR)	67 (61–73)
Male gender	65%
Smoking status	Current: 19%
	Former: 79%
	Never: 2%
Smoking history: median pack years (IQR)	48 (30–60)
Body mass index (kg/m^2^), median (IQR)	26.0 (23.0–29.1)
GOLD stage, % of patients	
1	8.70%
2	40.50%
3	32.90%
4	17.90%
GOLD risk class, % of patients	
A	4.00%
B	51.40%
C	8.70%
D	35.80%
Mean FEV_1_, % predicted (range)	51 (16–115)
Mean SGRQ score (range)	42 (5–83)
Mean mMRC score (range)	2 (0–4)
6MWD in metres, median (IQR)	418 (360–506)
BODE index, median (IQR)	3 (1–4)
Pharmacotherapy for COPD, % of patients	
SAMA	13%
SABA	39%
LAMA	86%
LABA	87%
ICS	54%
Systemic corticosteroids	8%
Mucolytics	10%
LTOT, % of patients	26%
CPAP, % of patients	7%
NIV, % of patients	9%
Supervised rehabilitation in the past, % of patients	31%
Volume reduction therapy, % of patients	7%
Comorbidities, % of patients	
Arterial hypertension	44%
Coronary artery disease	18%
Congestive heart failure	6%
Myocardial infarction	9%
Pulmonary hypertension	3%
Malignancy	9%
Diabetes	10%
Renal failure	8%
Psychiatric disease	9%
Influenza vaccination applied, % of patients	71%
Pneumococcal vaccination applied, % of patients	44%
Charlson comorbidity index, median (IQR)	1 (1–2)

CPAP, continuous positive airway pressure; FEV_1_, forced expiratory volume in 1 s; GOLD, Global Initiative for Obstructive Lung Disease; ICS, inhalative corticosteroids; IQR, interquartile range; LABA, long‐acting beta agonists; LAMA, long‐acting muscarinic antagonists; LTOT, long‐term oxygen therapy; NIV, noninvasive ventilation; SABA, short‐acting beta agonists; SAMA, short‐acting muscarinic antagonists.

## Primary end‐point – CAT

**Fig. 1:**
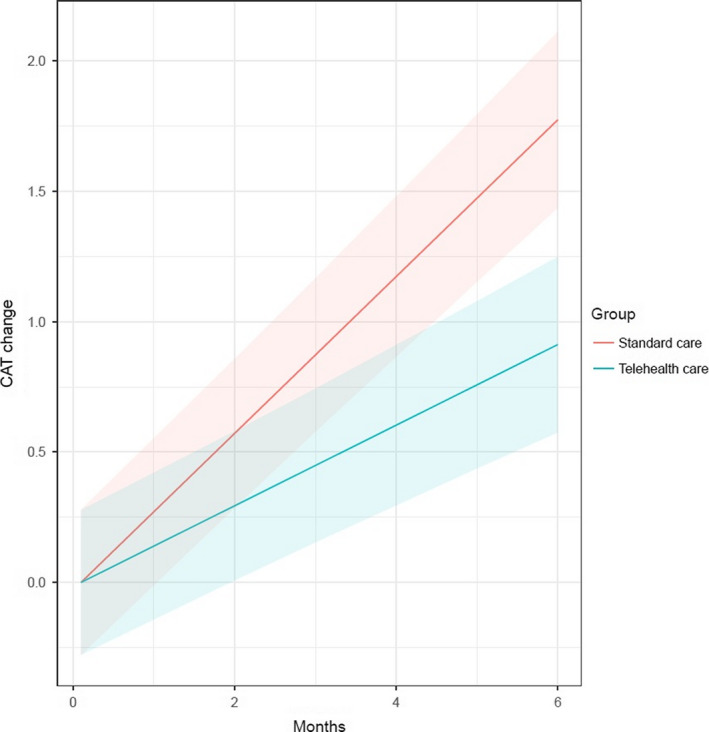
Mean CAT increase over time.

The overall mean CAT score was 15.6 points. The mean CAT increase was 1.8 (standard error 0.55) vs. 3.6 (standard error 0.56) points/year in the TC and SC groups, respectively (*P* = 0.0015; Figure 1).

## Secondary end‐point

Satisfaction with care on a visual analogue scale (ranging from 0 to 10) at baseline was 8.2; at the end of SC, 8.5 (mean of differences SC‐baseline = 0.26; 95% CI: −0.01 −0.53; *P* = 0.062); and after TC, 8.8 (mean of differences TC‐baseline = 0.64; 95% CI: 0.37–0.9; *P* < 0.001).

## Exploratory end‐points

The results of exploratory end‐points are summarized in Table 2.

**Table 2:** Exploratory end‐points

	TC	SC	*P*
ED visit rate due to AECOPD	0.47/year [95% CI: 0.37–0.59]	0.62/year [95% CI: 0.5–0.75]	0.140
Hospitalization rate due to AECOPD	0.17/year [95% CI: 0.11–0.25]	0.17/year [95% CI: 0.11–0.25]	0.618
Number of AECOPD (total)	125	99	0.060
Number of mild AECOPD	17	15	0.759
Number of moderate AECOPD	99	72	**0.028**
Number of severe AECOPD	9	12	0.524
Days in hospital due to AECOPD (total)	187	282	0.497
COPD‐related costs (total)	2812 USD/py	4609 USD/py	0.364
Mortality due to COPD (number)	0	0	–
Mortality overall (number)	0	5	–
Data completeness	88.2%	na	–
Number of phone contacts (total)	311	na	–
Duration of telephone contacts (mean, per py)	9.47 min	na	–

ED, emergency department min, minutes; na, not applicable; py, patient‐year; USD, US dollars.

## Discussion

Whilst receiving telehealth care, the slope of the CAT increase – an indicator of the naturally progressive course of COPD – was significantly reduced by 50% (−1.8 points/year) in comparison with standard care. Satisfaction with care was already high before the intervention and increased further with TC compared to SC. With TC, we detected a higher number of moderate AECOPD. TC was associated with a trend towards fewer days in hospital and lower total COPD‐related costs compared with SC.

Strength of this study is the multicentre, randomized controlled crossover design, so that every patient experiences both SC and TC, serving as his own control, minimizing selection bias and improving generalizability. The latter has also been supported by keeping inclusion criteria as open as possible and exclusion criteria at a minimum. By this, in fact, nearly all COPD patients were eligible for the study. To the best of the authors’ knowledge, the association between CAT values and telehealth interventions has not been investigated in such a manner until now.

The demonstrated bisection of the CAT increase over time reflects a highly relevant modification of the disease course of COPD. Looking at the above‐mentioned exploratory end‐points, one mechanism of how our TC procedure could have influenced disease course might be the better detection and consequently timely treatment of significantly more moderate exacerbations, which are underdiagnosed according to the literature [2]. Recently, Tupper *et al* [11] could not find a change in CAT score after 6 months of their TC approach. One difference to our study is that they measured CAT only at baseline and after the study, whereas we provide a weekly CAT and calculated the slope, resulting in a much higher resolution of values. Further, our TC approach systematically assessed patients on a daily basis, whereas the ‘reaction interval’ in the mentioned study was variable. This seems to be a critical factor for the success of telehealth interventions for COPD looking at other trials in that area, where we can observe that studies with longer reaction intervals tended to show negative results more frequently [12, 13]. In our view, another critical point besides reaction interval and mode of intervention is the choice of the primary end‐point. Many authors chose emergency department visits or hospitalizations and failed to show positive effects of their telehealth interventions [12, 13, 14, 15, 16, 17]. The main reason could be the fact that these events are rather rare, taking the whole COPD population into account, leading to the problem of underpowered studies. We made the same observation in our feasibility trial [9] and in this study (explorative end‐points).

Satisfaction with care significantly increased with TC, which we had already observed in a feasibility trial [9]. Our experience and a possible explanation were that patients appreciated the closer contact to the care team and the easier availability of support, for example by the possibility to make comments and ask questions using the TC platform. The increase in satisfaction with care by TC interventions has also been described by others [18, 19, 20].

Regarding total days in hospital due to COPD and total COPD‐related costs (including all costs related to the TC intervention), we could not find a statistically significant difference between TC and SC, but the actual numbers suggest that there might be a benefit by using TC. However, the current study was not powered to evaluate this hypothesis appropriately.

## Limitations

Regarding important exploratory end‐points like discussed above, we had insufficient statistical power to show significant differences. Achieving this would have required considerably more included patients.

## Conclusion

Whilst receiving TC, the slope of the CAT increase – an indicator of the naturally progressive course of COPD – was significantly reduced by 50% (−1.8 points/year). Satisfaction with care – already high before the intervention – increased further with TC. With TC, we detected a higher number of moderate AECOPD, probably indicating a higher diagnostic sensitivity than without TC. This could be achieved with manageable effort and by trend lower total COPD‐related costs.

## Conflict of interest statement

The study was funded by Swisscom Health AG, SWICA Krankenversicherung AG, PneumRx GmbH, AstraZeneca AG and Boehringer Ingelheim Schweiz GmbH. None of these had any influence on design, conduct, interpretation or writing of the manuscript. Prof. Kohler reports personal fees from Bayer, Boehringer, Novartis, Astra and Mundipharma, all outside the submitted work, and grants and personal fees from Roche and GSK. Prof. Tamm reports grants from Vifor AG and Schwabe Pharma AG, all outside the submitted work. Prof. Stolz reports grants from AstraZeneca AG, Curetis AG and Boston Scientific and personal fees from AstraZeneca AG, Novartis AG, GSK AG, Roche AG, Zambon, Pfizer, Schwabe Pharma AG and Vifor AG, all outside the submitted work.

## Author Contribution

**F. Rassouli:** Conceptualization (lead); data curation (lead); formal analysis (lead); funding acquisition (lead); investigation (lead); methodology (lead); project administration (lead); supervision (lead); visualization (lead); writing – original draft (lead); writing – review and editing (lead). **A. Germann:** Conceptualization (supporting); data curation (supporting); formal analysis (supporting); investigation (supporting); methodology (supporting); project administration (supporting); writing – original draft (supporting); writing – review and editing (supporting). **F. Baty:** Data curation (supporting); formal analysis (supporting); investigation (supporting); methodology (supporting); validation (supporting); visualization (supporting); writing – original draft (supporting); writing – review and editing (supporting). **M. Kohler:** Conceptualization (supporting); data curation (supporting); investigation (supporting); methodology (supporting); writing – original draft (supporting); writing – review and editing (supporting). **D. Stolz:** Conceptualization (supporting); data curation (supporting); investigation (supporting); methodology (supporting); writing – original draft (supporting); writing – review and editing (supporting). **R. Thurnheer:** Conceptualization (supporting); data curation (supporting); investigation (supporting); methodology (supporting); writing – original draft (supporting); writing – review and editing (supporting). **T. Brack:** Conceptualization (supporting); data curation (supporting); investigation (supporting); methodology (supporting); writing – original draft (supporting); writing – review and editing (supporting). **C. Kähler:** Conceptualization (supporting); data curation (supporting); investigation (supporting); methodology (supporting); writing – original draft (supporting); writing – review and editing (supporting). **S. Widmer:** Conceptualization (supporting); data curation (supporting); investigation (supporting); methodology (supporting); project administration (supporting). **U. Tschirren:** Data curation (supporting); investigation (supporting); project administration (supporting). **N. A. Sievi:** Data curation (supporting); investigation (supporting); project administration (supporting). **M. Tamm:** Conceptualization (supporting); data curation (supporting); investigation (supporting); methodology (supporting); writing – original draft (supporting); writing – review and editing (supporting). **M. H. Brutsche:** Conceptualization (supporting); data curation (supporting); formal analysis (supporting); funding acquisition (supporting); investigation (supporting); methodology (supporting); project administration (supporting); writing – original draft (supporting); writing – review and editing (supporting).

## Supporting information


**Figure S1**. Randomized crossover design (CAT: COPD assessment test; eCRF: electronic case report form).
**Figure S2**. Left upper part: patient view of the e‐health platform. By pressing “COPD study”, patients were transferred to the questionnaire. Lower part: screenshot of daily online questions to be answered by the patients (“yes” or “no”). Right upper part: “cockpit” of the study team with color‐coded alerts in the right column under “Status today” (red = AECOPD suspected, need for telephone call; yellow = more symptoms than usually, but for < 24 h; green = not more symptoms than usually; gray = questions not answered). Under “Weekly status”, the alerts of the last 7 days are displayed. Patients could make comments and ask questions. Adapted and translated from our pilot study [9] with permission from S. Karger AG, Basel, Switzerland.
