# Supplementary figures and images for: Telehealth mitigates COPD disease progression compared to standard of care: a randomized controlled crossover trial
Source: J Intern Med. 2021 Jan 11;289(3):404–10. doi: 10.1111/joim.13230 (PMC7986739; doi:10.1111/joim.13230)

**Online supplementary material**

Figure s1:


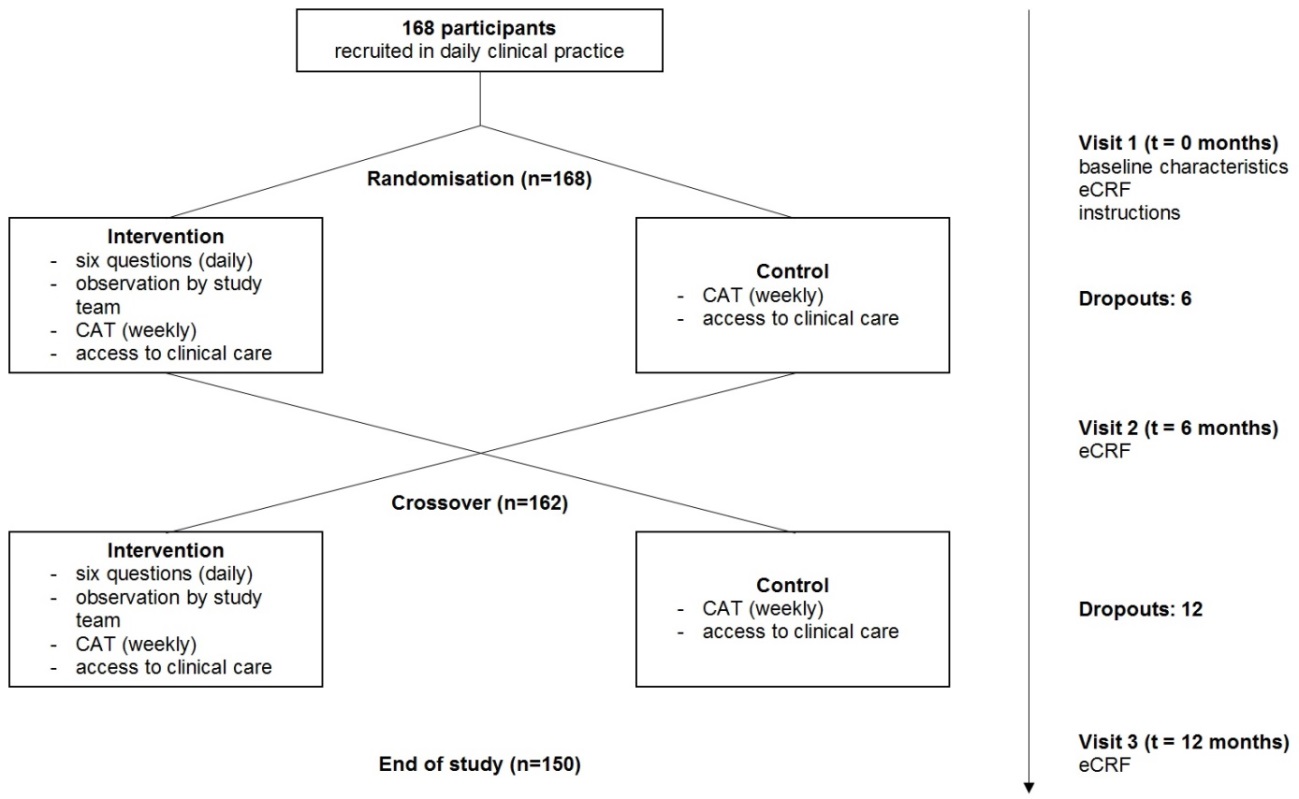


Figure s2:


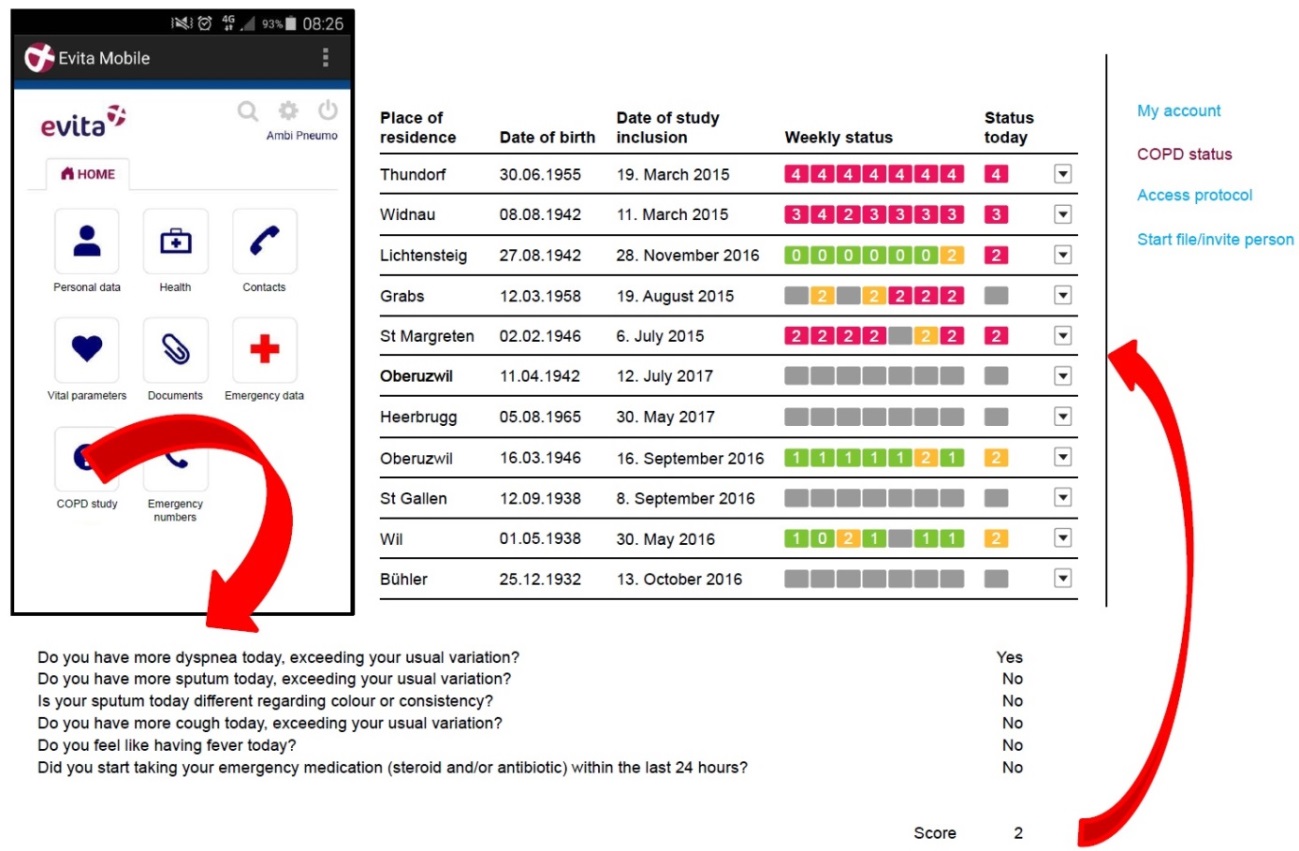

Supplement: Supplementary file 1 — Figure S1. Randomized crossover design (CAT: COPD assessment test; eCRF: electronic case report form). Figure S2. Left upper part: patient view of the e‐health platform. By pressing “COPD study”, patients were transferred to the questionnaire. Lower part: screenshot of daily online questions to be answered by the patients (“yes” or “no”). Right upper part: “cockpit” of the study team with color‐coded alerts in the right column under “Status today” (red = AECOPD suspected, need for telephone call; yellow = more symptoms than usually, but for < 24 h; green = not more symptoms than usually; gray = questions not answered). Under “Weekly status”, the alerts of the last 7 days are displayed. Patients could make comments and ask questions. Adapted and translated from our pilot study [9] with permission from S. Karger AG, Basel, Switzerland. [file JOIM-289-404-s001.docx]
